# Clinical Cases

**Published:** 1890-11

**Authors:** Clarence N. Payne

**Affiliations:** Bridgeport, Conn.


### CLINICAL CASES.

CLARENCE N. PAYNE, BRIDGEPORT, CONN.

RHEUMATISM.—Mrs. Emma K., colored, rheumatism. First saw patient May 28th, 1889. Found her in bed and that she had been sick two weeks, but would not have a doctor before, preferring to treat herself with all kinds of liniments, but as, in spite of this, she was getting worse, thought better of it and sent for me. Condition as follows: Pain appears in different joints and only stays in one place about two days, when it changes its location, as from ankle to knee, then hip, next the back, leaving parts first attacked only to return to them again.

Joints swollen, sore, and throbbing, worse by motion. Little thirst. Fair appetite. No sleep, because "so nervous." ℞ Puls.$^{15}$. Dose every two hours.

May 30th.—Better than any time for two weeks, yet weather is damp and rainy. Much less throbbing and pains, but joints stiff. No sleep. Continued Puls.$^{15}$.

June 1st.—Very little pain now, only stiffness on attempting to move, as if tendon pulled. Has had some sleep. ℞ Rhus$^{30}$. Two hours.

June 4th.—Stiffness better, but some return of soreness and pain. Returned to Puls.$^{15}$.

June 6th.—Found patient walking about out-doors for first time. Discharged cured.

VOMITING AND DIARRHŒA.—Susan C., age twenty-one.

Called June 21st, 1889. *History:* For past three weeks has vomited nearly everything taken on stomach. Region of stomach very sore to pressure. Eructations tasting of food. Has been chilly and hot alternately for past three weeks. Has kept about, however, till to-day. Has had more or less attention from an old-school physician with no good result.

*Present condition* as above, and as follows: Bowels loose, small, thin, green movements. Pain in back and limbs, so severe cannot keep still, must move. Seems like a nervous agitation. Severe headache, worse on top. Tongue yellow and dry. Poor sleep last two weeks. Water will *stay* on stomach a *few moments* and is *then vomited.*

Temperature 103.8°. Pulse 120. ℞ Phos.$^3$.

June 22d.—Found patient had not vomited after taking Phos. Stomach feels better. Pain in head and limbs still very severe. Restless and poor sleep. No record of temperature and pulse this day. Changed remedy to Rhus$^3$.

June 23d.—Much better in nearly every respect. Less restless. Some sleep. All pain much relieved. More *soreness* of bowels, however, which are tympanitic, and soreness worse on right side, low down, bowels moving often, greenish-yellow, but without pain or tenesmus. Noticed, for first time, a red triangular tip on tongue, the remainder of tongue having yellow coating. Temperature fallen to 100.3°, and pulse to 88. Rhus$^{30}$.

June 24th.—Pain all gone. Temperature and pulse *normal.* No nausea or vomiting. Tenderness of bowels better. Bowels still moving, but without pain and more natural color. ℞ China$^3$.

As patient was eight miles in country, I did not see her again, but on June 27th she sent word that she was rapidly convalescing.

Diarrhœa.—Elmer B., age about twenty-one, blacksmith. Called to patient July 21st, 1890. Found him in condition of great prostration. For past three nights (especially) had many profuse watery stools, preceded by colicky pain and rumbling, and imperative desire for stool, followed by sense of emptiness in bowels and relief of pain. Has also much nausea, vomiting, and retching. Intense thirst for large amounts. Tongue coated with red tip and edges. Gave him Verat-alb.$^3$ which immediately controlled whole condition. No return of movements or vomiting after first dose.

